# Public Health Impact of Coal and Electricity Consumption: Risk–Benefit Balance Varies by Country

**DOI:** 10.1289/ehp.119-a258a

**Published:** 2011-06

**Authors:** Tanya Tillett

**Affiliations:** **Tanya Tillett**, MA, of Durham, NC, is a staff writer/editor for *EHP*. She has been on the *EHP* staff since 2000 and has represented the journal at national and international conferences

Access to electricity contributes to good health by powering infrastructure for clean drinking water and sanitation and by reducing the need for indoor burning of coal, wood, and other solid fuels. But these benefits can be offset by health threats posed by the emissions from fossil fuel–based electricity production—direct public health effects attributable to particulate matter, sulfur and nitrous oxides, volatile organic compounds, carbon monoxide, and ozone are estimated to account for more than 70% of the external costs of power generation (i.e., costs not factored into the price paid for electricity). A multitiered analysis of the relationship between coal consumption, electricity use, and health outcomes uses three complementary data sets to compare positive and negative health effects of power generation **[*EHP*** **119(6):821–826; Gohlke et al.]**.

The authors developed an autoregressive time-series model of infant mortality, life expectancy, electricity consumption, and coal consumption for 41 different countries over the period 1965–2005. They divided the countries into three groups depending on infant mortality and life expectancy rates as of 1965: countries with high infant mortality and low life expectancy, those with moderately high infant mortality and medium to high life expectancy, and those with low infant mortality and high life expectancy.

Model predictions suggested infant mortality would decrease with increasing electricity consumption over time, but only in countries that started off with high infant mortality and low life expectancy, a group that included Algeria, Brazil, India, Indonesia, Pakistan, Peru, South Africa, and Turkey. Models did not predict a change in life expectancy with increased electricity use, but did predict a decrease in life expectancy with increased coal consumption in countries with moderate infant mortality and life expectancy in 1965. In addition, infant mortality was predicted to increase with increased coal consumption in those countries with low infant mortality and high life expectancy.

The authors compared these results with estimates from two independent methods for modeling health effects of energy-related environmental exposures. The first method, the World Health Organization’s Environmental Burden of Disease model, estimates the burden of human disease related to outdoor air pollution, indoor air pollution, drinking water, and sanitation. The second method, the Greenhouse Gas and Air Pollution Interactions and Synergies model developed by the International Institute for Applied Systems Analysis, estimates potential life-shortening effects of pollutant emissions from coal-fired power plants. Estimates from both models were consistent with those derived from the authors’ autoregressive model.

The study’s limitations include a lack of comprehensive data for variables such as education level, vaccination rates, and health care access and/or expenditures. However, the consistency of the results of the three analyses strongly supports the authors’ conclusions and highlights ways that human health impacts might be integrated into climate change mitigation and energy policy research.

## Figures and Tables

**Figure:**
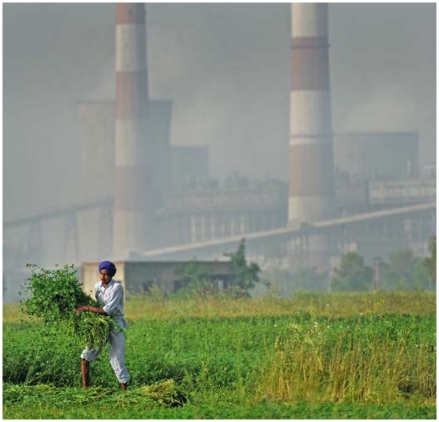
Outside the coal-fired Guru Hargobind Thermal Power Station, Bathinda, Punjab, India

